# Hospital Notes

**Published:** 1891-07

**Authors:** 


### Hospital Notes.

The Buffalo State Hospital graduated its fifth class of nurses from the training school connected with that institution on Tuesday, June 16, 1891, consisting of nine women and five men. The opening of the new and spacious ward on the west side of the administration building was also celebrated at the same time. It was an interesting day, and Dr. J. B. Andrews may be well pleased with the results of his first ten years' labor as Superintendent of this splendid hospital.

---

The Buffalo General Hospital Training School for Nurses held its thirteenth annual graduating exercises on Tuesday, June 23, 1891, at which nine young women were duly commissioned, and certified as competent to nurse and care for sick and suffering humanity. There is no loftier mission for young women than this, and if, perchance, it becomes a shorter route to matrimony, as it has been attempted to show is the case by some statistical fiend, what of it? All the better for the patient, all the better for the nurse. Let the next class enter!

